# Novel Insight into Mutational Landscape of Head and Neck Squamous Cell Carcinoma

**DOI:** 10.1371/journal.pone.0093102

**Published:** 2014-03-25

**Authors:** Daria A. Gaykalova, Elizabeth Mambo, Ashish Choudhary, Jeffery Houghton, Kalyan Buddavarapu, Tiffany Sanford, Will Darden, Alex Adai, Andrew Hadd, Gary Latham, Ludmila V. Danilova, Justin Bishop, Ryan J. Li, William H. Westra, Patrick Hennessey, Wayne M. Koch, Michael F. Ochs, Joseph A. Califano, Wenyue Sun

**Affiliations:** 1 Department of Otolaryngology-Head and Neck Surgery, Johns Hopkins Medical Institutions, Baltimore, Maryland, United States of America; 2 Asuragen Inc., Austin, Texas, United States of America; 3 Department of Pathology, Johns Hopkins Medical Institutions, Baltimore, Maryland, United States of America; 4 Department of Oncology and Health Science Informatics, Johns Hopkins Medical Institutions, Baltimore, Maryland, United States of America; 5 Milton J. Dance Head and Neck Center, Greater Baltimore Medical Center, Baltimore, Maryland, United States of America; Winship Cancer Institute of Emory University, United States of America

## Abstract

Development of head and neck squamous cell carcinoma (HNSCC) is characterized by accumulation of mutations in several oncogenes and tumor suppressor genes. We have formerly described the mutation pattern of HNSCC and described NOTCH signaling pathway alterations. Given the complexity of the HNSCC, here we extend the previous study to understand the overall HNSCC mutation context and to discover additional genetic alterations. We performed high depth targeted exon sequencing of 51 highly actionable cancer-related genes with a high frequency of mutation across many cancer types, including head and neck. DNA from primary tumor tissues and matched normal tissues was analyzed for 37 HNSCC patients. We identified 26 non-synonymous or stop-gained mutations targeting 11 of 51 selected genes. These genes were mutated in 17 out of 37 (46%) studied HNSCC patients. Smokers harbored 3.2-fold more mutations than non-smokers. Importantly, TP53 was mutated in 30%, NOTCH1 in 8% and FGFR3 in 5% of HNSCC. HPV negative patients harbored 4-fold more TP53 mutations than HPV positive patients. These data confirm prior reports of the HNSCC mutational profile. Additionally, we detected mutations in two new genes, CEBPA and FES, which have not been previously reported in HNSCC. These data extend the spectrum of HNSCC mutations and define novel mutation targets in HNSCC carcinogenesis, especially for smokers and HNSCC without HPV infection.

## Introduction

HNSCC is a disease with significant morbidity and mortality. It is the fifth most common cancer, which is responsible for 5% of all tumor patients, and accounts for 560,000 new cancer incidents and 300,000 cancer deaths worldwide [1]. More than 50,000 new cases of HNSCC are diagnosed in the United States yearly, with a mortality rate of 12,000 annually [2]. The survival rate is only 50% within 5 years after diagnosis [2]. This malignancy is highly related to habitual factors, such as smoking, alcohol consumption, and infection with human papilloma virus (HPV), which has been associated with the majority of oropharynx cancers [3], [4], [5]. Despite the advances in medical care and operative skills, mortality of HNSCC has not been significantly improved for the past three decades. Therefore investigation of the molecular biology of HNSCC can strongly enhance the development of modern chemotherapy agents, which will improve and prolong the lifespan of HNSCC patients.

Like other solid and smoking-related tumors, HNSCC develops via accumulation of multiple genetic and epigenetic alterations [6], [7], [8]. There are several critical steps during HNSCC development, such as progressive allelic loss of 9p, 3p, 17p, 11q, 3q, 14q, 6p, 8p, 4q, accommodated by mutation and inactivation of CDKN2A, multiple mutations of TP53, mutations in and amplification of CDH1 and inactivation of pTEN [9], [10], [11], [12], [13]. Later data demonstrated additional mutation events in PIK3CA/PIC3R1, RB1, and EGFR [14], [15], [16], [17]. Discovery of the overexpressed EGFR due to EGFR high polysomy and gene amplification in HNSCC led to the development and application of EGFR-specific cetuximab-based immunotherapy for HNSCC treatment [18], [19].

To acquire the comprehensive genetic signature of HNSCC, two independent groups performed next generation targeted exon sequencing of tumor DNA and matched normal DNA for 32 [20] and 74 [21] HNSCC patients. Both groups confirmed mutation rate in the genes that were previously reported for HNSCC, including TP53, CDKN2A, PIK3CA, PTEN, and HRAS. Importantly both groups reported the mutations in NOTCH1 for the first time in HNSCC. Overall they have demonstrated 14–15% mutation rate for NOTCH1. Later on NOTCH1 was sequenced and mutations in this gene were confirmed for head and neck cancer cell lines [22]. Mutations in the genes of PI3K pathway were also further validated [16]. Additional new mutated genes in HNSCC were discovered by both Agrawal et al and Stransky et al [20], [21].

In order to validate the discovery of NOTCH mutations in HNSCC and to evaluate the role of NOTCH pathway in HNSCC tumorigenesis we recently performed a comprehensive analyses of the genetic, epigenetic and transcriptional alterations of NOTCH pathway in a cohort containing 44 HNSCC tumor and 25 normal tissues [23]. HNSCC tissues were deep-sequenced for NOTCH1 mutation and demonstrated 10.8% mutation rate of this gene, similar to previous reports [20], [21]. We also demonstrated a bimodal pattern of NOTCH pathway alterations in HNSCC, while NOTCH1 receptor is targeted by inactivating mutations in 10.8% of HNSCC, the other 32% of HNSCC have significant upregulation of NOTCH pathway, determined by ligand and receptor activation, expression, and copy number increase [23].

In this work we extend our initial analysis of HNSCC mutational landscape, and annotate additional genetic alteration events in HNSCC. We employ next generation sequencing techniques to profile samples from 37 HNSCC patients from Johns Hopkins University. Based on our prior HNSCC mutation reports and publications reporting highly mutated genes in different tumor types, we select 51 highly actionable cancer related genes, including TP53, EGFR, HRAS, SRC, ABL1, PI3K, and others [3], [6], [9], [12], [15], [16], [20], [21], [24], [25], [26]. These 51 genes were selected from COSMIC, the Catalogue Of Somatic Mutations In Cancer, based upon the spectrum of mutations in individual cancer types with established or potential role in HNSCC tumorigenesis [27]. As a result, this study allows us to gain a comprehensive understanding of the established and additional abnormal genetic alterations in HNSCC.

The deep sequencing analysis that was performed in this work allows us to successfully confirm the mutation rates for HNSCC-related genes, such as TP53, NOTCH1, CDH1 and PIK3CA genes. We also report here the association of smoking and increased mutation rates. Moreover, 38% of the point mutations discovered in this study have not been previously reported in the COSMIC database [20], [21], [27]. New genes, CEBPA and FES, are identified to be mutated in HNSCC. These are known tumor suppressor (CEBPA) and proto-oncogene (FES) that have been shown to be mutated in several tumor types [28], [29], [30]. Adaptation of targeted therapy for the mutated forms of these genes has the potential to improve clinical outcome of individual HNSCC patients carrying such mutations.

## Materials and Methods

### Tissue samples

Primary tumor tissues and matched lymphocytes were collected from 37 HNSCC patients at Johns Hopkins Hospital. Every participant signed a written informed consent before participating in this study. This study was approved by Johns Hopkins Medicine Internal Review Board (JHM IRB), and performed under approved research protocol NA_00036235. All samples were stored at −140°C (liquid nitrogen) until use. All cancer samples were examined by board certified Pathologists from the Pathology Department of Johns Hopkins Hospital, JHH (WHW and JB). Tumor samples confirmed as HNSCC were subsequently microdissected to yield at least 75% tumor content. The clinical characteristic of the study cohort is listed in Table 1. These samples were adopted from a previously reported discovery cohort, used for analysis by multiple high-throughput platforms including DNA copy number, methylation, and expression analysis [23].

**Table 1:** Clinical characteristics of the cohort.

	HNSCC (n = 37), n (%)
Median age (range)	58±12 (34–87)
Male	26 (70%)
Female	11 (30%)
Race	
Caucasian	35 (95%)
African American	2 (5%)
Smoking status	
Pack-years (range)	25.9±31.5 (15–125)
Smokers	24 (65%)
Non-smokers	8 (22%)
Unknown	5 (13%)
Drinking status	
Drink	21 (57%)
Do not drink	9 (24%)
Unknown	7 (19%)
HPV16 positive	9 (24%)
Tumor site	
Oral cavity	8 (22%)
Oropharynx	13 (35%)
Larynx	12 (32%)
Hypopharynx	4 (11%)
TNM stage	
I	4 (11%)
II	2 (5%)
III	5 (14%)
IV	26 (70%)
Disease status	
No evidence of disease	18 (49%)
Alive with disease	1 (3%)
Dead of disease	15 (40%)
Dead of unrelated causes	3 (8%)

### DNA preparation

Microdissected tissue samples and collected lymphocytes were digested in 1% SDS (Sigma) and 50 μg/ml proteinase K (Invitrogen) solution at 48°C for 48–72 hours for removal of proteins bound to DNA. DNA was then purified using standard phenol-chloroform extraction and ethanol precipitation methods as previously described [31], [32]. DNA was resuspended in LoTE buffer (EDTA 2.5 mM and Tris-HCl 10 mM, pH 7.5). FFPE DNA was isolated from the enriched FFPE sections using the RecoverAll Total Nucleic Acid Isolation Kit (Ambion) according to the manufacturer's instructions. DNA concentration was quantified using the NanoDrop ND-1000 spectrophotometer (Thermo Scientific).

### Content selection for targeted exon next-generation sequencing

The SuraSeq® 7500 enrichment array was developed for deep sequencing analysis across many cancers and included a total of 51 genes selected from the COSMIC mutation database v58 (see Table 2 for details). Many of these genes, such as TP53, HRAS, RB1, PI3K and other have been previously shown to be mutated in many tumors, including HNSCC [9], [16], [20], [21]. Other genes, such as ABL1, GATA1, PAX5 are involved in key cancer-related pathways, and known to be mutated in other tumor types, including leukemia, pancreatic and lung cancer [27], [33], [34], [35], [36]. Expression of several genes, including HIF1A, is known to be altered in HNSCC, but their mutational status was never evaluated. Twenty-eight of the selected genes were analyzed by targeted “hotspot” sequencing. These include genes in which mutations preferentially occur in specific loci (hot spots) and they were selected for targeted sequencing in those specific regions. Such regions contained predominantly single nucleotide substitutions within the selected exons. Twenty-three more genes with a more distributed mutational profile across many exons, such as tumor suppressors, were selected for sequencing of entire coding exons (whole exon sequencing), covering 85–100% of the coding sequence. Refer to Table 2 for more details.

**Table 2:** The 51 cancer genes selected for targeted or whole exon sequencing.

#	Gene Symbol	Selected cancers with mutations	Reports in HNSCC	Selected pathways	Selected ref.	Sequencing analysis
1	ABL1	CML; ALL; T-ALL	none	ATM signaling pathway; cell cycle	[33], [43]	targeted exon
2	AKT1	breast; ovarian; NSCLC	none	Apoptosis and DNA damage	[44], [45]	targeted exon
3	AKT2	ovarian; pancreatic	yes	Apoptosis; AMPK signaling	[46], [47]	targeted exon
4	BRAF	melanoma; colorectal; NSCLC	yes	MAP Kinase signaling pathway	[48], [49]	targeted exon
5	CDH1	lobular breast; gastric	yes	TGF beta signaling pathway	[10], [21]	whole exon
6	CDK4	melanoma	none	P53 signaling pathway	[50]	targeted exon
7	CDKN2A	melanoma; pancreatic; HNSCC	yes	Apoptosis modulation and signaling	[20], [51]	whole exon
8	CEBPA	AML; MDS	none	EGFR signaling pathway	[30]	whole exon
9	CREBBP	ALL; AML; DLBCL; B-NHL	yes	IL-7 signal transduction; DNA damage	[21], [52]	targeted exon
10	CTNNB1	colorectal; hepatoblastoma	none	Wnt and TGFB Signaling Pathways	[53], [54]	whole exon
11	EGFR	glioma; NSCLC; HNSCC	yes	EGF signaling pathway	[18], [21]	whole exon
12	ERBB2	breast; ovarian; NSCLC; gastric	yes	ERBB signaling pathway	[20], [55]	whole exon
13	FES	bladder; colon; lymphoma	none	GF and cytokine receptor signaling	[28], [29]	targeted exon
14	FGFR1	MPD; NHL	none	FGF signaling pathway	[56]	whole exon
15	FGFR3	bladder; MM; T-cell lymphoma	yes	FGF signaling pathway	[21], [41]	targeted exon
16	FLT3	AML; ALL	yes	Hematopoietic cell lineage	[21], [57]	targeted exon
17	FOXL2	ovarian granulosa-cell tumor	none	Ovarian development and function	[58]	targeted exon
18	GATA1	megakaryoblastic leukaemia	none	Hemostasis; erythrocyte differentiation	[59], [60]	targeted exon
19	GNA11	uveal melanoma	none	Peptide induced signaling pathway	[61]	targeted exon
20	GNAQ	uveal melanoma	yes	G protein signaling pathway	[21], [62]	targeted exon
21	HIF1A	breast; renal; pancreatic	none	Homeostatic response to hypoxia	[63], [64]	whole exon
22	HRAS	rhabdomyosarcoma; bladder	yes	Signal transduction pathways	[20], [65]	targeted exon
23	IDH1	glioblastoma	none	Krebs cycle	[66]	targeted exon
24	IDH2	glioblastoma	none	Krebs cycle	[66]	targeted exon
25	IKBKB	leukemia; lymphoma; kidney	none	NF-kb signaling pathway	[67]	targeted exon
26	JAK2	ALL; AML; MPD; CML	yes	JAK/STAT signaling pathway	[16], [68]	targeted exon
27	KIT	GIST; AML; TGCT; melanoma	yes	Hematopoiesis; melanogenesis	[21], [69]	whole exon
28	KRAS	pancreatic; colorectal; thyroid	yes	Signal transduction pathways	[21], [65]	whole exon
29	MEN1	parathyroid; pancreatic	none	Chromatin remodeling	[70], [71]	whole exon
30	MET	papillary renal; HNSCC	yes	HGF signal transduction pathways	[43], [72]	targeted exon
31	MPL	MPD	yes	TPO signaling pathway	[20], [73]	targeted exon
32	NF2	meningioma; acoustic neuroma	yes	Hippo signaling pathway	[21], [74]	whole exon
33	NOTCH1	T-ALL; HNSCC	yes	NOTCH signaling pathway	[20], [21]	whole exon
34	NPM1	NHL; APL; AML	none	Centrosome duplication	[75]	targeted exon
35	NRAS	melanoma; MM; AML; thyroid	yes	Signal transduction pathways	[65]	targeted exon
36	PAX5	NHL; ALL; B-ALL	none	Transcription regulation	[76]	targeted exon
37	PDGFRA	GIST; paediatric glioblastoma	none	GF signal transduction pathways	[77]	targeted exon
38	PIK3CA	colorectal; gastric; glioblastoma	yes	PI3K/AKT signaling pathway	[16], [20]	targeted exon
39	PIK3R1	glioblastoma; ovarian; HNSCC	yes	PI3K/AKT signaling pathway	[16], [21]	whole exon
40	PTCH1	medulloblastoma	yes	Hedgehog signaling pathway	[20], [78]	whole exon
41	PTEN	glioma; prostate; endometrial	yes	PI3K/AKT signaling pathway	[11], [21]	whole exon
42	PTPN11	JMML; AML; MDS	none	Signal transduction pathways	[79], [80]	targeted exon
43	RB1	retinoblastoma; sarcoma; SCLC	yes	Cell cycle check point	[21], [81]	whole exon
44	RET	thyroid; pheochromocytoma	yes	Signal transduction pathways	[21], [82]	targeted exon
45	SMAD4	Pancreatic; colorectal; HNSCC	yes	Signal transduction pathways	[20], [83]	whole exon
46	SMARCB1	malignant rhabdoid	none	SWI/SNF chromatin-remodeling	[84]	whole exon
47	SMO	skin basal cell	yes	Hedgehog signaling pathway	[21], [85]	whole exon
48	SRC	colon, liver, lung, breast	none	Signal transduction pathways	[86], [87]	targeted exon
49	STK11	NSCLC; pancreatic	yes	Signal transduction pathways	[88], [89]	whole exon
50	TP53	majority of cancer types	yes	Cell cycle regulation	[12], [20]	whole exon
51	VHL	renal; haemangioma	none	Signaling pathways	[90]	whole exon

### Target enrichment

Exon sequencing was performed by Asuragen, Inc. (Austin, Texas). Gene-specific primers were designed to amplify products up to 200 bp. Genomic DNA from HNSCC tumor tissues and matched lymphocytes, used as a putative germline reference, was fragmented to an average size of ∼4 kb using the Covaris S220 (Covaris, Woburn, MA). All DNA samples were evaluated for the extent of fragmentation following analysis using E-gels (Life Technologies). Fragmented genomic DNA (500 ng) was then merged with an emulsified ∼2000 member primer library (SuraSeq® 7500) using the RainDance RDT 1000 platform. The RDT 1000 instrument is a microfluidic chip-based platform that incorporates microdroplet-based technology to amplify hundreds to thousands of genomic loci with high specificity and uniformity [37]. Templates within merged droplets were amplified using the following PCR conditions: 1 cycle of 94°C for 2 min, 55 cycles of (94°C for 15 sec; 54°C for 15 sec; 68°C for 30 sec), 1 cycle of 68°C for 10 min and 4°C hold. Following emulsion breaking, the resulting PCR products were purified using Qiagen MinElute kit according to the manufacturer's instructions. A fraction of the purified PCR products was examined for size and quantity using the Agilent Bioanalyzer Lab-on-a-chip DNA 12000 and the Nanodrop, respectively.

### Deep sequencing with the Illumina GAIIx platform

Following gene-specific PCR, a tagging PCR reaction was performed to append unique barcode sequences to the gene-specific products from each sample and to add adapters specific for sequencing on the Illumina GAIIx platform. Samples were fingerprinted for multiplex sequencing using one of 48 barcodes (Illumina). Purified products (10 ng) were tagged using the following conditions: 1 cycle of 94°C for 2 min, 10 cycles of (94°C for 30 sec; 56°C for 30 sec; 68°C for 1 min), 1 cycle of 68°C for 10 min, and 4°C hold. These PCR products were then pooled and purified using Qiagen MinElute PCR purification kit, and quantified using the KAPA Library Quant kit (KAPA Biosystems, Cape Town, South Africa) following the manufacturer's instructions. All samples were normalized to 8.6 nM and pools of 15 samples per lane were prepared. Flow cell preparation and data acquisition were completed using Illumina's recommended protocols. Paired-end sequencing runs (2×151) were performed using the Illumina GAIIx platform.

### Mutation Confirmation on MiSeq

Samples from patient X15 and X33 were selected for targeted confirmation sequencing on the MiSeq, spanning custom amplicons. All sequencing was performed by Asuragen. Gene-specific primers were designed to amplify targeted products up to 106 bp, with fully assembled products (containing adapter sequences compatible for the MiSeq) up to 233 bp. Templates were amplified as previously described [38]. For each sample, PCR products with Illumina adapters and barcodes (5 μL) were purified and normalized using 5 μL of AxyPrep™ Mag PCR Normalization and Clean-Up System, according to the manufacturer's recommendation (Axygen, Union City, California). Samples were eluted with 25 μL (EB-N) and equal volumes (2 μL) were pooled to make a single multiplex library. The pooled library was quantified using the KAPA Library Quantification Kit following the manufacturer's instructions. The library was then denatured and prepared to 13.6 pM in the presence of 15% PhiX (2.4 pM) to increase base diversity. Flow cell preparation and sequencing on the MiSeq was performed using version 3 chemistry, as recommended by the manufacturer's protocol.

### Somatic mutation calling

The sequence read data generated from the GAIIx were demultiplexed, adapter and primer sequences were removed and trimmed to retain high quality data (q20 or higher) [37], [39], [40]. The sequence read data were filtered, aligned and variant scores calculated as previously described [38]. We retained only the high coverage regions for analysis, where high coverage was defined as having greater than 2% of the sample median coverage and above 100 reads. We also flagged the loci that are known to be associated with systematic positives. For each set of matched samples, we filtered out variants that were present in the lymphocytes as putative germline variants, or as sample-specific systematic error. In our analysis, if the matched normal loci were found to have greater than 1% variant reads, or the variant score difference was within the 99.5% percentile of all pair-wise differences for non-annotated loci across the matched pair, the variant was removed from consideration. The used mutation read frequency threshold, 6%, has positive prediction value with over 90% confidential threshold. Variants were annotated using gene structure from snpEff version 2.1b. Coding base substitutions are classified as missense, nonsense, splice site, or silent. All sequencing data are available in NCBI bioProject database. The SRA Accession Number is SRR1055850

### Statistical analysis

P-values were calculated using Fisher exact test, comparing mutation event or mutation rate with clinical characteristics.

## Results

### Clinical characteristics of the 37 HNSCC tumors

To discover HNSCC genetic signature and new genetic mutations, we have performed selected deep exon sequencing on 37 HNSCC tumors and their paired normal lymphocytes (Table 1).

The characteristics of the HNSCC population largely reflect the demographics of head and neck cancer patients in the United States (Table 1). The HNSCC patients were largely male (70%, 26 of 37) and Caucasian (95%, 35 of 37), age 34 to 87 years (median ± SD  =  58±12 years). There was a history of tobacco and alcohol consumption in 65% (24 of 37) and 57% (21 of 37) of all patients, respectively, with average smoking history being 25.9 pack-years (range: 4-125 packs per year). With regard to HPV status, the study population consisted of 24% (9 of 37) HPV-positive patients. Primary tumors were located in the oral cavity (22%, 8 of 37), oropharynx (35%, 13 of 37), hypopharynx (11%, 4 of 37), or larynx (32%, 12 of 37). Twenty-six of 37 patients (70%) presented with locally advanced stage IV disease. The median follow-up time of these patients was 31.4 months (range: 0.5–117.3 months). At the end of the follow-up period, 10 patients were alive with disease. As of September 2013, a total of 18 patients (48%) have died. The cause of death was head and neck cancer in 15 out of 18 patients; the other three patients died of unrelated causes.

### Mutation spectrum in 37 HNSCC tumors

**Figure 1:**
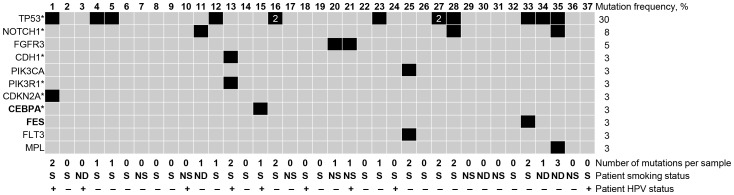
Genetic alterations in 37 HNSCC tumors. Heat-map representation of individual mutations present in a series of 37 HNSCC tumors, presented in columns. Mutation events are represented by black color. Left, Mutated genes, asterisks indicate genes characterized by whole-exon sequencing. Novel genes mutated in HNSCC are labeled by bold font. Right, mutation rate for each gene. Genes are ranked by mutation rate. Bottom, number of mutations per tumor sample. Note, that two patients have two mutations in the same gene TP53 gene: X16 and X27 (with number 2 on the heat-map). The smoking status of tumor patients is identified as: S – for smokers, NS – for never smoked patients, ND – not determined. The HPV status of tumor patients is identified as: “+” for HPV-positive patients and “-” for HPV-negative patients.

In our analysis, we have found a total number of 26 disruptive mutations detected in 46% (17 of 37) HNSCC patients (Figure 1). These mutations include 21 non-synonymous and 5 stop-gained mutations (Table 3) that were detected in 11 out of the 51 genes being sequenced, including p53, NOTCH1, and CDH1. We have also detected other mutated genes, such as PIK3CA, PIK3R1, CDKN2A, and FGFR3, which were previously implicated in HNSCC tumorigenesis [13], [16], [21]. Of note, 10 of the detected 26 site-specific point mutations (38%) were never reported in the COSMIC database for any cancer type [27].

**Table 3:** Complete list of point mutations among the 11 cancer genes sequenced in the 37 HNSCC tumor tissues.

#	Gene Symbol	AA COSMIC	Chromosome	Position	Reference Base	Alternate Base	Effect	CDS Change	Prior AA Reports	Sample ID
1	CDH1	p.I96T	16	68835696	T	C	NON_SYNONYMOUS	aTc/aCc	None	X19
2	CDKN2A	p.G130V	9	21971012	C	A	NON_SYNONYMOUS	gGa/gTa	None	X1
3	CEBPA	p.R323G	19	33792354	G	C	NON_SYNONYMOUS	Cgc/Ggc	None	X15
4	FES	p.S96L	15	91428715	C	T	NON_SYNONYMOUS	tCa/tTa	None	X33
5	FGFR3	p.K652N	4	1807891	G	T	NON_SYNONYMOUS	aaG/aaT	None	X21
6	FGFR3	p.S249C	4	1803568	C	G	NON_SYNONYMOUS	tCc/tGc	Yes	X20
7	FLT3	p.F590L	13	28608286	G	C	NON_SYNONYMOUS	ttC/ttG	None	X25
8	MPL	p.L500V	1	43814963	C	G	NON_SYNONYMOUS	Cta/Gta	None	X35
9	NOTCH1	p.E1294*	9	139401189	C	A	STOP_GAINED	Gag/Tag	None	X35
10	NOTCH1	p.R56*	9	139418406	G	A	STOP_GAINED	Cga/Tga	None	X28
11	NOTCH1	p.W1843*	9	139396309	C	T	STOP_GAINED	tgG/tgA	Yes	X11
12	PIK3CA	p.E545K	3	178936091	G	A	NON_SYNONYMOUS	Gag/Aag	Yes	X25
13	PIK3R1	p.M26I	5	67588148	G	A	NON_SYNONYMOUS	atG/atA	None	X13
14	TP53	p.C135F	17	7578526	C	A	NON_SYNONYMOUS	tGc/tTc	Yes	X1
15	TP53	p.R175H	17	7578406	C	T	NON_SYNONYMOUS	cGc/cAc	Yes	X27
16	TP53	p.C176Y	17	7578403	C	T	NON_SYNONYMOUS	tGc/tAc	Yes	X4
17	TP53	p.H179R	17	7578394	T	C	NON_SYNONYMOUS	cAt/cGt	Yes	X33
18	TP53	p.Y220C	17	7578190	T	C	NON_SYNONYMOUS	tAt/tGt	Yes	X5
19	TP53	p.Y234N	17	7577581	A	T	NON_SYNONYMOUS	Tac/Aac	Yes	X16
20	TP53	p.Y234S	17	7577580	T	G	NON_SYNONYMOUS	tAc/tCc	Yes	X12
21	TP53	p.S241F	17	7577559	G	A	NON_SYNONYMOUS	tCc/tTc	Yes	X16
22	TP53	p.R248Q	17	7577538	C	T	NON_SYNONYMOUS	cGg/cAg	Yes	X34
23	TP53	p.R273C	17	7577121	G	A	NON_SYNONYMOUS	Cgt/Tgt	Yes	X28
24	TP53	p.R282W	17	7577094	G	A	NON_SYNONYMOUS	Cgg/Tgg	Yes	X23
25	TP53	p.Q317*	17	7576897	G	A	STOP_GAINED	Cag/Tag	Yes	X35
26	TP53	p.R342*	17	7574003	G	A	STOP_GAINED	Cga/Tga	Yes	X27

Overall, TP53 was found mutated 13 times in 29.7% (11 of 37) HNSCC patients, and FGFR3 was found mutated in 5.4% (2 of 37) HNSCC patients (Figure 1 and Table S1). NOTCH1 mutations were found in 8.1% (3 of 37) patients, similar to prior reports [20], [21], [23]. Together with mutated genes that were previously identified and reported for HNSCC, we have discovered new mutation events for HNSCC. The novel mutated genes include: CEBPA and FES (Table 3 and S3). Other mutations can be found in Figure 1 and Tables 3 and S1.

We validated the CEBPA and FES mutations in matched FFPE tumor DNA using the SuraSeq 7500 enrichment panel, and also in frozen tumor tissue using a targeted amplicon PCR based library preparation couple with sequencing on the MiSeq platform (Illumina). Confirmatory analysis yielded similar results with 46% and 14% read frequency for CEBPA and FES mutations in samples X15 and X33, respectively (Table S3). The high mutation read frequency rate, 29% (CEBPA) and 30% (FES), was detected in FFPE DNA, confirming our original discovery. FES mutation was also validated by the analysis of HNSCC-TCGA data and demonstrated 1.4% mutation rate of this gene in HNSCC (Table S1).

### Correlation with clinical data

We evaluated the association of mutations with clinical characteristics, such as tumor site, tumor stage, tumor recurrence, patient gender, race, age, HPV infection, follow-up period, disease status, smoking, and alcohol consumption status. We have found correlation of mutation status with smoking. As expected, more mutations were identified in tumor samples from patients with a history of tobacco consumption: 0.25 mutations in non-smoking group vs. 0.79 mutations in smoking group on average. Thus, at least one mutation was found in 50% of smoking patients vs. only 25% of non-smoking patients (Figure 1). Similarly, 71% of patients with somatic tumor mutations were found to be smokers, while no more than 60% of patients without any detected mutations in our study had history of tobacco use. Interestingly, there were no mutations detected in the commonly mutated TP53, NOTCH1, and CDH1 genes within the non-smoking group from our study. In general there were only 2 mutation events in the non-smoking group both in FGFR3 gene. Interesting, no FGFR3 mutations were found in the smoking population.

In agreement with previous reports [20], [21], [26] we have found increased TP53 mutation rate in the HPV negative population. On average the HPV-negative population has a TP53 mutation rate that is 4-foles lower that TP53 mutation rate in the HPV-positive population, 0.43 and 0.11 mutations, respectively. No other correlations of clinical characteristics with mutation status were detected in our study group.

## Discussion

### HNSCC mutational landscape

HNSCC is a complex disease, usually characterized by accumulation of genetic and epigenetic alterations [7], [8], [9], [20], [21]. In this work, we have performed a comprehensive next generation exon sequencing of 51 tumor suppressor genes, oncogenes, currently actionable genes, and potentially actionable genes, that were selected based on frequency of mutation across many cancer types, including HNSCC. We were able to support our prior discovery of HNSCC mutations, as well as to discover new genetic alterations in HNSCC. Moreover, among the mutated 11 genes, that were previously shown mutated in HNSCC and/or other tumor types, we have discovered new sites of point mutations for 38% of them. In order to see the general picture of the mutation events, we have drafted the common pathways including discovered 11 genes (Figure S1). The majority of the mutations were found in trans-membrane receptors or in the signal transduction pathways, such as PI3K/AKT and Wnt/CDH. The other group of mutated genes belongs to transcription factors (Figure S1)

### Correlation with the previous data

Our data are highly consistent with previously published reports from our group and other investigators. Thus, TP53 was found mutated in 47% [20] in 62% [21] and in 42% of HNSCC tumor samples, as reported by COSMIC [27]. Our analysis detected 30% of HNSCC samples harbored TP53 mutations (Figure 1 and Table S1). The lower rate of the detected TP53 mutations may be explained by the fact that the 5′UTR and 3′UTR of TP53 were not covered by the selected panel. Agrawal and Stransky groups have reported 14% and 15% mutations in NOTCH1 [20], [21]. We have supported their data and have noted 8.1% mutation rate for NOTCH1. Stransky and colleagues [21] also found 2 mutations of CDH1 in 2.7% patients, matching our results. PIK3CA was found mutated in 4.1% to 10.6% of HNSCC patients [16], [21]. We were able to detect PIC3CA in one tumor sample (2.7%). Similar rate of mutation was found for PIK3R1, 2.6% in [16] and 2.7% in our work. Our results are in agreement with the data from Stransky and colleagues showing low incident (5.4%) of FGFR3 mutations in HNSCC population (1.35% in [21]). On the other hand, the G2128T (pG697C) mutation of FGFR3 was previously found in 62% of OSCC [41]. However, the question of FGFR3-pG697C being a polymorphism, rather than a mutation remains unclear.

We have included several genes into deep-sequencing analysis, which were previously reported in several tumor types, but not in HNSCC. These are ABL1, AKT1, CEBPA, FOXL2, GATA1, IDH1, IDH2, VHL and more (Table 2). Two of them were found mutated in our study in HNSCC for the first time: CEBPA and FES.

### Novel mutations

CEBPA and FES single mutations were detected in our experiments. The incidence of these novel mutations cannot be accurately ascertained due to the sample size. Higher rate of mutation for those genes could be confirmed with a larger sample size.

CEBPA, CCAAT/enhancer-binding protein alpha, is a tumor-suppressor gene. Its activity was diminished by the multiple mutations found in different tumor sites [30]. The mutated fraction for CEBPA was as high as 44% (Tables S2 and S3). We have further confirmed this mutation in a matched FFPE tumor DNA sample for this novel mutation in HNSCC. This G-to-C mutation was detected in FFPE DNA at 29%. We have further validated this novel mutation in CEBPA through ultra-deep sequencing of the frozen tumor DNA using Illumina MiSeq platform (Table S3). Interestingly, pR323G mutation found in CEBPA protein lies within highly mutated Leucine zipper domain. Argenine at the position 323 was never shown to be substituted by mutations in any other cancer types, including cervix cancer and leukemia [27].

In case of non-receptor protein-tyrosine kinase FES, pS96L mutation is a new addition to the mutation library for this protein. It lies just outside of FCH domain that allows binding to cytoskeleton. Such mutation was never found for any other cancer types, even among esophageal and lung adenocarcinomas, known for FES mutations [29], [42]. Both the SuraSeq 7500 enrichment panel and the confirmatory assays on the MiSeq platform detected the C-to-T mutation at 12% and 14%, respectively. This mutation was also detected in the matched FFPE tumor sample at 30%. 279 HNSCC patient cohort of TCGA detected FES mutation with 1.4% mutation rate (Table S1), but none of TCGA cohort mutations targeted S96 amino acid.

### Mutation rate and smoking

Among the 37 HNSCC patients participated in this study 24 (65%) have history of tobacco use. The smoking population of this study carries the majority (73%) of all detected mutations with 0.79 mutations per sample, which is 3.2-fold higher than number of mutations in non-smoking group (22%, 8 out of the 37 HNSCC). The non-smoking group bearing overall 2 mutations each found in 2 out of 8 patients. Both mutations targeted FGFR3, not found mutated in any smoking patient in our study. The other 6 non-smoking patients did not have any detected mutations. The additional 5 patients from our study group did not specify their smoking history. Interestingly, within the smoking group, the mutation was as high as 37.5% for TP53. None of the newly discovered mutations in CEBPA and FES genes were found in the non-smoking group.

### TP53 mutations and HPV

Overall 30% of studied HNSCC samples harbor TP53 mutation. This rate is lower than previously reported TP53 mutation rate in COSMIC (42%) and in our prior report (47% in [20]), see Table S1 for details. The reason why such a low incidence (30%) of TP53 mutations found in this cohort is unclear. We had nine HNSCC patients with HPV infection in oropharynx out of total 37 samples in our study group. Only one of them harbor TP53 mutation, similar to results in [26]. Other reports could not detect any TP53 mutations in HPV-positive samples, which could be explained by the underrepresentation of HPV-positive samples in their cohorts [20], [21]. In agreement with these prior reports [20], [21], [26] we detected higher TP53 mutation rate in HPV-negative patients: 0.43 in HPV-negative vs 0.11 in HPV-positive patients, While other reports demonstrated higher rate of TP53 mutations in HPV-negative group: from 73% to 100%, we have found only the modest number, 37.5%, of HPV-negative patients to harbor TP53 mutation (Figure 1). This fact can be explained by the fact that we did not analyze 5′UTR and 3′UTR of TP53.

### Summary

Using targeted exon sequencing in primary HNSCC, we have validated and confirmed prior published mutational data, and identified 26 point mutations in 11 out of 51 analyzed genes. Although most of the mutated genes have been described in other solid tumors, mutations in two of them have not been previously detected in HNSCC. Among these 11 genes, 4 and 8 were also found mutated in the studies from our group and Stransky group, respectively. Of note, 10 out of the 11 genes were found mutated in the studies from TCGA group. The common genes identified mutations in our studies and the other three studies included TP53, NOTCH1, and PIK3CA. Yet only gene mutations in 10 out of 11 reported genes were found recurrently mutated in these three studies. Agrawal's group reported 3 out of 11 genes to be recurrently mutated: TP53, NOTCH1 and PIK3CA. Stransky discovered these three as well as CDKN2A, CDH1 and PIK3R1 genes to be recurrently mutated. On the other hand, among the 26 non-synonymous or stop-gained mutations: 12 were found in other three studies mentioned above and only 6 of them were found recurring in these three studies. Of note, CEBPA was not previously reported in any cited HNSCC sequencing studies, and was further thoroughly validated in our study (including MiSeq ultra-deep validation and FFPE DNA sequencing re-evaluation). Altogether, low frequency of the mutations was recurring in HNSCC and reflects extent of tumor heterogeneity. The mutation rate and functional consequence of newly discovered mutated genes in HNSCC will be further investigated.

The role of newly discovered mutated genes, as well as the role of novel point mutations in previously reported genes, requires further validation using a larger HNSCC cohort and understanding of their role in HNSCC development and metastasis. The utilization of the newly adopted Asuragen SuraSeq® 7500 platform for deep sequencing analysis of additional FFPE DNA samples will be further investigated. The mutation status of additional genes, including FBXW7, Caspase8, and Fat-1 [20], [21] in HNSCC will be also investigated in the prospective study. Such insight is particularly needed for HNSCC populations with a prior or current history of smoking and without HPV infection. In the future, some of these novel mutations identified here may be considered for personalized targeted therapies in HNSCC patients.

## Supporting Information

Figure S1 **Pathways alterations in HNSCC.** 11 genes with detected mutations were used to draft simplified pathways, altered in HNSCC. Several trans-membrane receptors, transcription factors and members of signal transduction domains were found mutated during this study. The PI3K/AKT and Wnt/CDH1 pathways were altered through several mechanisms. The rate of mutation for each individual gene is reported below the gene name. Red, blue and green color stands for oncogenes, tumor suppressor genes and for genes with dual function, respectively.
(TIF)

Table S1 **Frequency of mutations in 11 genes for 37 HNSCC tissues.**
(XLSX)

Table S2 **Read depth and frequency for the detected 26 mutations.** Mutation rate prevalence was calculated by dividing mutation frequency in tumor by mutation frequency in matched normal tissue. N/D is for not determined, due to the 0 number of the mutated calls in matched normal tissue.
(XLSX)

Table S3 **Validation of CEBPA and FES mutations in frozen tissues and FFPE slides.**
(XLSX)
