# Correction: Novel Insight into Mutational Landscape of Head and Neck Squamous Cell Carcinoma

**DOI:** 10.1371/journal.pone.0233409

**Published:** 2020-05-13

**Authors:** Daria A. Gaykalova, Elizabeth Mambo, Ashish Choudhary, Jeffery Houghton, Kalyan Buddavarapu, Tiffany Sanford, Will Darden, Alex Adai, Andrew Hadd, Gary Latham, Ludmila V. Danilova, Justin Bishop, Ryan J. Li, William H. Westra, Patrick Hennessey, Wayne M. Koch, Michael F. Ochs, Joseph A. Califano, Wenyue Sun

There is an error in the first row of Table 3. Please see the correct Table 3 here.

**Table 3:** Complete list of point mutations among the genes sequenced in the 37 HNSCC tissues.

#	Gene Symbol	AA COSMIC	Chromosome	Position	Reference Base	Alternate Base	Effect	CDS Change	Prior AA Reports	Sample ID
1	CDH1	p.G382D	16	68847223	G	A	NON_SYNONYMOUS	gGt/gAt	None	X13
2	CDKN2A	p.G130V	9	21971012	C	A	NON_SYNONYMOUS	gGa/gTa	None	X1
3	CEBPA	p.R323G	19	33792354	G	C	NON_SYNONYMOUS	Cgc/Ggc	None	X15
4	FES	p.S96L	15	91428715	C	T	NON_SYNONYMOUS	tCa/tTa	None	X33
5	FGFR3	p.K652N	4	1807891	G	T	NON_SYNONYMOUS	aaG/aaT	None	X21
6	FGFR3	p.S249C	4	1803568	C	G	NON_SYNONYMOUS	tCc/tGc	Yes	X20
7	FLT3	p.F590L	13	28608286	G	C	NON_SYNONYMOUS	ttC/ttG	None	X25
8	MPL	p.L500V	1	43814963	C	G	NON_SYNONYMOUS	Cta/Gta	None	X35
9	NOTCH1	p.E1294*	9	139401189	C	A	STOP_GAINED	Gag/Tag	None	X35
10	NOTCH1	p.R56*	9	139418406	G	A	STOP_GAINED	Cga/Tga	None	X28
11	NOTCH1	p.W1843*	9	139396309	C	T	STOP_GAINED	tgG/tgA	Yes	X11
12	PIK3CA	p.E545K	3	178936091	G	A	NON_SYNONYMOUS	Gag/Aag	Yes	X25
13	PIK3R1	p.M26I	5	67588148	G	A	NON_SYNONYMOUS	atG/atA	None	X13
14	TP53	p.C135F	17	7578526	C	A	NON_SYNONYMOUS	tGc/tTc	Yes	X1
15	TP53	p.R175H	17	7578406	C	T	NON_SYNONYMOUS	cGc/cAc	Yes	X27
16	TP53	p.C176Y	17	7578403	C	T	NON_SYNONYMOUS	tGc/tAc	Yes	X4
17	TP53	p.H179R	17	7578394	T	C	NON_SYNONYMOUS	cAt/cGt	Yes	X33
18	TP53	p.Y220C	17	7578190	T	C	NON_SYNONYMOUS	tAt/tGt	Yes	X5
19	TP53	p.Y234N	17	7577581	A	T	NON_SYNONYMOUS	Tac/Aac	Yes	X16
20	TP53	p.Y234S	17	7577580	T	G	NON_SYNONYMOUS	tAc/tCc	Yes	X12
21	TP53	p.S241F	17	7577559	G	A	NON_SYNONYMOUS	tCc/tTc	Yes	X16
22	TP53	p.R248Q	17	7577538	C	T	NON_SYNONYMOUS	cGg/cAg	Yes	X34
23	TP53	p.R273C	17	7577121	G	A	NON_SYNONYMOUS	Cgt/Tgt	Yes	X28
24	TP53	p.R282W	17	7577094	G	A	NON_SYNONYMOUS	Cgg/Tgg	Yes	X23
25	TP53	p.Q317*	17	7576897	G	A	STOP_GAINED	Cag/Tag	Yes	X35
26	TP53	p.R342*	17	7574003	G	A	STOP_GAINED	Cga/Tga	Yes	X27
