# Supplementary figures and images for: Novel Insight into Mutational Landscape of Head and Neck Squamous Cell Carcinoma
Source: PLoS One. 2014 Mar 25;9(3):e93102. doi: 10.1371/journal.pone.0093102 (PMC3965530; doi:10.1371/journal.pone.0093102)

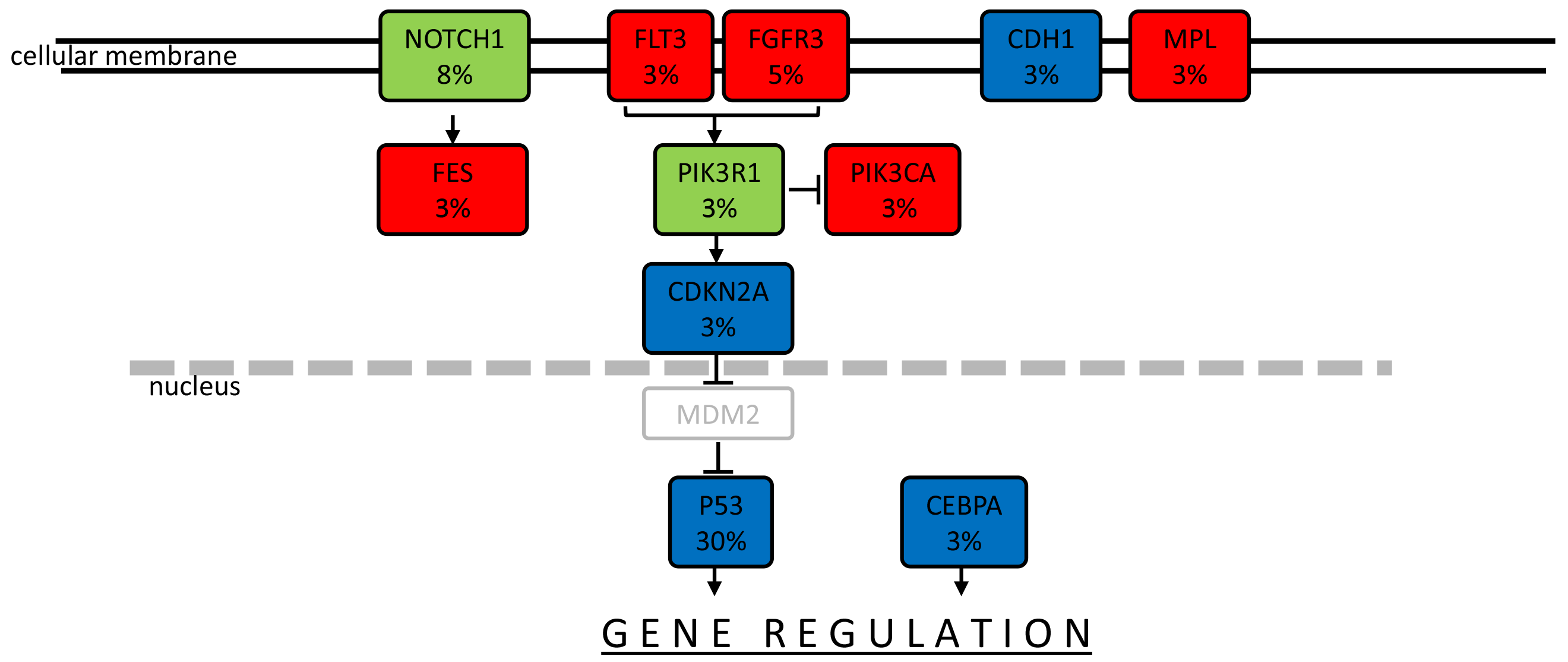

Supplement: Figure S1 — Pathways alterations in HNSCC. 11 genes with detected mutations were used to draft simplified pathways, altered in HNSCC. Several trans-membrane receptors, transcription factors and members of signal transduction domains were found mutated during this study. The PI3K/AKT and Wnt/CDH1 pathways were altered through several mechanisms. The rate of mutation for each individual gene is reported below the gene name. Red, blue and green color stands for oncogenes, tumor suppressor genes and for genes with dual function, respectively. (TIF) [file pone.0093102.s001.tif]
